# Psychedelic Treatment with Psilocybin: Addressing Medical Malpractice Risk and Physicians’ Concerns

**DOI:** 10.1017/jme.2025.10109

**Published:** 2025

**Authors:** Katherine Cheung, Maxwell Brodie, Sue-Ling Chang, Pierre Deschamps, Jean-Sébastien Fallu, Houman Farzin, Johanne Hébert, Jean-François Stephan, Michel Dorval, Yann Joly, for the P3A Study Group

**Affiliations:** 1 National Institutes of Health, Bethesda, United States; 2 McGill University Centre of Genomics and Policy, Montreal, Canada; 3 Axe Oncologie, Centre de recherche du CHU de Québec-Université Laval, Canada; 4 Independant author, Canada; 5 Universite de Montreal, Montreal, Canada; 6 Jewish General Hospital, Montreal, Canada; 7 McGill University, Montreal, Canada; 8 Department of Nursing Sciences, Universite du Quebec a Rimouski, Rimouski, Canada; 9 Centre de recherche du CISSS de Chaudière-Appalaches, Canada; 10 Institut universitaire de santé mentale de Montréal, Canada; 11 Universite Laval Faculte de Pharmacie, Québec City, Canada; 12 Axe Oncologie, Centre de recherche du CHU de Québec, Canada

**Keywords:** psilocybin, malpractice, liability, psychedelic treatment, psilocybin-assisted therapy, negligence

## Abstract

Psychedelic treatment with psilocybin is receiving increased attention following clinical trials showing it may help treat end-of-life anxiety, depression, and several other conditions. Despite this, physicians may be reluctant to prescribe psilocybin and carry out psilocybin treatment because of the stigma surrounding psychedelics and the potential for medical malpractice liability. This paper explores whether psilocybin treatment gives rise to a risk of medical malpractice liability for physicians. Following an overview of psilocybin treatment and its regulatory regime in Canada, exploratory vignettes are used to highlight the relevance and limits of malpractice claims. This paper argues that the lack of established medical standards, standardized training, and credentialing contribute to liability risks surrounding psilocybin treatment. More clinical trials, meta-studies of research analyses, and knowledge sharing will help to develop training programs and medical standards of practice to better realize psilocybin’s potential.

## Introduction

The medical and psychotherapeutic use of psilocybin, often referred to as psilocybin treatment and psilocybin-assisted therapy, has been receiving increased attention in Canada from researchers, patients, physicians and the general public.^1^ Positive preliminary data obtained from clinical trials suggests that psilocybin may help treat end-of-life anxiety and depression, as well as several other conditions.^2^ However, even when permitted by law, it is uncertain whether most physicians are ready to prescribe psilocybin to patients or welcome the clinical use of psilocybin treatment. Physicians’ reluctance to consider psilocybin as a valid medical alternative to more traditional therapies, such as antidepressants, may be associated with the stigma around the use of psychedelic drugs and the perception that their involvement in psilocybin treatment could generate unacceptable risks of medical malpractice claims against them.

Medical malpractice liability is a field of law developed to compensate for harm suffered by victims of medical errors and to deter health care providers from engaging in substandard and unreasonable health care practices. In an exploratory survey of 119 psychologists in the United States, 51% answered that medical liability concerns would decrease their likelihood of informing patients about psilocybin treatment, or to refer them to someone with more expertise in this domain.^3^ This article aims to explore the potential contexts in which medical liability risks may appear in psilocybin treatment and, when possible, identify important considerations that would promote good medical practice in this context. As medical malpractice laws may vary from one country to another, we have focused primarily on the Canadian legal regime, including both the common law provinces and Quebec’s civilian system. Nevertheless, other legal regimes governing this issue share sufficient similarities for our manuscript to also be of interest to researchers, policymakers, and lawyers in other countries. Similarly, while this paper focuses on the role and liability of the physician in psilocybin treatment, much of the analysis will be relevant to other medical professionals involved. Many of the general remarks on liability will also be relevant to other psychedelic treatments.

## Psilocybin: History, Characteristics, and Clinical Use

### Evolving Social Trends on the Use of Psychedelic Therapy in the Medical Context

Psilocybin is currently experiencing a surge in popularity as part of a “psychedelic renaissance,” with an increasing number of people, including the public, physicians, and researchers showing interest in exploring the potential of psychedelics for treating various mental health disorders. In recent years, there has been a significant increase in the number of published journal articles on psilocybin and in the number of clinical trials conducted. Interest from the private sector is correspondingly growing, with numerous firms looking to develop their own psychedelic molecules.^4^ This “renaissance” comes after a period of inactivity and prohibition that followed promising, yet highly controversial, experiments with psychedelic drugs in the 1950s.^5^ Notably, it is at that time that Canadian researchers pioneered several studies on psychedelics, with Saskatchewan-based psychiatrist Humphry Osmond coining the word “psychedelic” in a letter to Aldous Huxley in 1956.^6^ While the 1960s saw the greatest amount of psychedelic research of the mid-twentieth century, cultural attitudes toward psychedelics began shifting, with psychedelics increasingly perceived as dangerous compounds due to their link with the “hippie counterculture.” Moreover, their association with the “War on Drugs” and the student anti-war demonstrations in the United States subsequently led to a more aggressive governmental stance against them in the late 1960s, with their eventual strict prohibition in most countries, including Canada.^7^

The past two decades have seen a resurgence in clinical and public interest in the neurobiological effects and therapeutic potential of psychedelics. In Canada, the first legal psychotherapeutic use of psilocybin was approved in August 2020 through a section 56 exemption to the Controlled Drugs and Substances Act (CDSA), exempting pharmacists, practitioners, and patients involved in that application from the CDSA prohibition.^8^ More recently, in 2022 the Canadian Institutes of Health Research (CIHR) issued the first federal grant to conduct a clinical trial on psilocybin treatment for treatment-resistant depression, end-of-life distress, and substance use disorders,^9^ with several other privately funded studies currently underway.^10^

The increase in medical assistance in dying (MAiD) requests in Canada has also brought psilocybin treatment into the spotlight. With end-of-life distress being a known reason for pursuing MAiD, and psilocybin having shown positive effects on end-of-life distress and treatment-resistant depression, many are pushing for psilocybin treatment to be accorded greater attention.^11^ In February 2023, Parliament’s Special Joint Committee Report on MAiD recommended reviewing programs and policies to improve access to promising alternative therapies and explicitly mentioned psilocybin as one treatment worthy of greater consideration.^12^ With the recent Bill C-7 expanding MAiD beyond situations where natural death is foreseeable, health professionals should consider informing patients of all means available to relieve their suffering.^13^ This may further promote research and clinical use of psilocybin treatment and influence the willingness of physicians to inform patients about this alternative treatment.

### Scientific Evidence of Clinical Utility and Safety

Given the infancy of psilocybin treatment as a field, evidence regarding the clinical utility and safety of it is still in the process of being established. However, in recent clinical trials conducted, psilocybin has been found to significantly improve conditions targeted, such as treatment-resistant depression, anxiety, and substance use disorders.^14^ Studies, albeit with small sample sizes, have shown a substantial improvement in symptoms within a week of psilocybin administration to patients with treatment-resistant depression.^15^ Clinical improvement often persists for a relatively long period of time after a single administration of psilocybin, with effects lasting from 4 weeks to up to 12 months.^16^ In addition to its antidepressant and antianxiety effects, Agin-Liebes et al. found that psilocybin administration was associated with long-term spiritual well-being and life satisfaction.^17^ Similarly, Erritzoe et al. found that psilocybin administration was associated with increased openness and cognitive flexibility in treatment-resistant depression patients.^18^ However, most studies on psilocybin are limited by their small sample size and difficulty with blinding, with larger, randomized controlled trials needed in the future to allow for more generalizable results.

Although larger studies are also needed to help provide confirmation of the safety of psilocybin treatment, thus far no verifiable deaths have been reported and serious adverse events (e.g. suicidal ideation or self-injury) are rare in supervised clinical studies.^19^ Regarding the risk of adverse reactions, researchers have found psilocybin to have a low potential to create dependence and addiction.^20^ Within clinical trials, psilocybin is generally well-tolerated, with mild psychological and physical adverse effects transitorily occurring, the most common being an elevated heart rate, mild emotional distress, nausea and headaches.^21^ The potential for severe adverse effects seem to be mitigated by the use of careful exclusion criteria and the controlled setting of clinical trials.^22^ However, it should be remembered that exclusion criteria in clinical studies can be narrower than clinical practice, sometimes because of the motivation to ensure that the study has a greater chance of obtaining publishable results.

Nonetheless, some people may still have a negative experience. Outside of the clinical setting, a survey of almost 2,000 people who have used psilocybin highlighted that 8% found their most negative experience decreased their well-being and life satisfaction, 11% reported putting themselves or others at risk of harm, and 10% showed negative symptoms (such as fear, anxiety, and depression) persisting more than a year after using psilocybin.^23^ There has also been at least one accepted case of hallucinogenic persisting perception disorder (HPPD), consisting of recurrent perceptual disturbances similar to experiences following consumption of the psychedelic, lasting for several years.^24^ While the frequency of negative outcomes following psilocybin consumption will likely be lower in the clinical setting,^25^ the fact that many eligible psilocybin treatment participants are already in a state of vulnerability and poor health (such as severe depression) could exacerbate their susceptibility to harm.

### A Psilocybin Session

In evaluating the risk of medical liability, it may help to examine the framework within which a psilocybin treatment session currently takes place to see how errors, oversights, and damages may arise. Before patients can access psilocybin treatment, they are carefully screened using exclusionary criteria looking for factors such as a history of bipolar disorder or psychosis.^26^ As for the therapeutic process, psilocybin therapy generally has two phases in addition to the actual psilocybin experience: a preparatory phase and an integrative phase.

Before the psilocybin experience, the preparatory phase takes place, where the therapist helps the patient prepare for the experience and engages with their expectations, securing informed consent, usually throughout several sessions. A therapeutic relationship is established during this period, in addition to mutual respect and trust.^27^ After this process is complete, the patient undergoes the supervised psilocybin session. With a typical session lasting between six and eight hours, a psychiatrist is rarely present the entire time. Typically, a psychiatrist oversees therapists (often two),^28^ with these therapists monitoring and guiding the patient through the experience.^29^ Should the patient experience distress during the experience, practitioners of psilocybin therapy may incorporate reassuring touch (also known as therapeutic or supportive touch) where it has been discussed in advance of the session with the patient during the informed consent process.^30^ Reassuring touch can include a touch on the arm or shoulder as well as holding the participant’s hand.

After the psilocybin session, the primary therapist and patient will “examine, process, and integrate the psychedelic experience” in the integrative phase, thereby helping the patient to find and attribute meaning to the experience.^31^ Academics have noted that the integration phase is not precisely defined and can look different across providers, leading to a confusion as to how best to practice it. A range of models of the integration phase have been identified, each associated with different worldviews (Indigenous and/or Western psychological), audiences (therapists, facilitators broadly, etc.), and scope (number of sessions, comprehensiveness of discussion).^32^

## Psilocybin in Canada Policy Framework

### Laws and Regulations Applicable to the Therapeutic Use of Psilocybin in Canada

In Canada, psilocybin is designated as a “controlled substance” under the CDSA and is classified under Schedule 3.^33^ Passed in 1996, the CDSA provides a legislative framework to address potentially dangerous drugs and fulfill Canada’s international obligations under the United Nations Single Convention on Narcotic Drugs.^34^ Under the CDSA, all activities (sale, production, possession) involving psilocybin and its psychoactive derivative, psilocin, are illegal unless authorized by Health Canada, the federal department responsible for administering the CDSA and issuing licenses, exemptions and guidance pursuant to it. Psilocybin is additionally classified as a drug under the Food and Drugs Act.

However, in October 2022, the province of Alberta amended its provincial Mental Health Services Protection Regulation to regulate psychedelic therapy, including using psilocybin, MDMA, LSD, ketamine, and other drugs. These amendments came into effect in January of 2023. Clinics are now able to provide psychedelic therapy, provided they obtain a license, among other requirements laid out in Alberta’s framework.^35^

Currently, Alberta is the only province in Canada that permits psilocybin treatment. In other provinces, those who are eligible can access psilocybin using one of three methods: through clinical trials, an exemption recognized under Section 56 of the CDSA, or through the Special Access Program under the Food and Drug Regulation*s.* Given the scientific interest in psilocybin, Health Canada released a notice in May 2022 that emphasized the importance of conducting clinical trials for drugs like psilocybin that have not received market authorization. The notice highlighted that clinical trials are the most effective method to gather scientific data and knowledge about these drugs, with the ultimate goal of advancing their approval process.^36^ However, clinical trials maintain strict inclusion/exclusion criteria and may present logistical challenges for participants living outside of large urban areas, making it an inaccessible option for many.^37^

As an alternative to clinical trials, individuals may also be granted a Section 56 exemption under the CDSA. The CDSA states that the Minister may “exempt from the application of all or any of the provisions of this Act or the regulations any person or class of persons or any controlled substance or precursor or any class of either of them if, in the opinion of the Minister, the exemption is necessary for a medical or scientific purpose or is otherwise in the public interest.”^38^ Under this program, patients submit an application with the support of their physician. However, wait times for a response are highly variable (from 48 hours to 300 days), and patients must obtain the psilocybin themselves which could encourage the use of unregulated and illicit sources.^39^ It is worth noting that Quebec became the first province to financially cover psilocybin treatment when it reimbursed a section 56 exempted psilocybin session in December 2022.^40^

The third method that individuals may pursue to access psilocybin treatment is through the Special Access Program, recently expanded in January 2022 to include psilocybin.^41^ The Special Access Program (SAP) allows healthcare professionals to access drugs that are not yet authorized for sale in Canada for patients with serious or life-threatening conditions.^42^ Regulatory amendments to the Food and Drugs Act at the end of 2021 restored the possibility for physicians to request access to restricted drugs (such as psilocybin) through the SAP.^43^ The SAP enables a decision to be made; however, substantial paperwork must still be completed by the physician requesting psilocybin, and patients not falling within the predefined criteria are automatically excluded.^44^ To make a request, a healthcare professional completes a lengthy form made available by Health Canada detailing patient information, practitioner information, clinical rationale, drug information, past treatment, among other factors. This request is then triaged based on time sensitivity.^45^ Overall, all currently available methods of accessing psilocybin in Canada have drawbacks for patients. Those who are ineligible to participate in clinical trials or who may not have a doctor willing to apply through the SAP may not be able to access psilocybin treatment.

In a recent lawsuit before the Federal Court, the refusal of an SAP access request to psilocybin to treat cluster headaches was deemed unreasonable by the Court. According to this judgment, the delegate of the Minister of Health had not meaningfully addressed the key issues brought forward by the patient and his doctor, failing to consider the Charter arguments that they had raised in their request.^46^ Consequently, the SAP access request was remitted for a different Delegate of the Minister of Health to reassess. The Court made it clear that substantial justifications provided in support of the request would need to be appropriately addressed and that a swift decision on the part of the Delegate was expected. This is a positive message to the Minister of Health and its delegates that future SAP access requests should not be turned down lightly. However, a second court case before the Superior Court challenging the Minister of Health’s decision to deny access to psilocybin to various healthcare professionals for their own professional training for psilocybin therapy was dismissed as the applicants failed to demonstrate that the Minister’s refusal was unreasonable.^47^

In the US context, the argument for a constitutional right for terminally ill patients to access experimental drugs was rejected by a majority of the DC Circuit Court of Appeals sitting *en banc* in 2007.^48^ Nevertheless, the American court noted that arguments in favour of broadening access to experimental therapies “are certainly ones that can be aired in the democratic branches” (leading to Congressional action a decade later).^49^ Looking forward, clinical evidence, developing medical standards, raising awareness, and a better understanding of the legal risks for practitioners will be central in efforts to broaden access to psilocybin treatment in both the Canadian and American contexts.

### Standards of Practice

In new areas of medicine that can be perceived as controversial by stakeholders, the emergence of reliable standards for physicians can act as key enablers promoting up-to-date and safe medical practice.^50^ Psilocybin can bring about profound changes in a patient’s consciousness and may lead to long-term personality changes, as well as cause serious adverse effects, such as psychosis.^51^ Furthermore, the heightened suggestibility and vulnerability of patients during psilocybin sessions underscores the need for clear ethical guidelines to prevent any inappropriate behavior by therapists.^52^ Providing evidence-based medical and ethical standards can promote patients’ well-being and confidence in psilocybin treatment. Standards also provide useful guidance for physicians working in this nascent area to quell fears of malpractice liability and reputational damage. This, in turn, can lead to a greater level of comfort and increased willingness of physicians to inform qualifying patients about psilocybin treatment. Discussions in the two recent Canadian court cases summarized in the previous section highlighted the importance of clinical standards and guidelines on psilocybin treatment for assessing an SAP access request.^53^

It is agreed upon that clinical practice guidelines should be based on the best scientific evidence,^54^ with the Institute of Medicine (IOM)’s 2011 standards for generating clinical practice guidelines being an often referred to standard.^55^ The American Psychological Association and IOM both typically develop guidelines in two steps: “(a) the conduct of a comprehensive systematic review of the empirical literature that is presented to a guideline development panel (GDP) and (b) evaluation of the findings from the systematic review by the panel that considers the quality of evidence and the relative benefits and harms associated with the clinical practices reviewed.”^56^

However, due to the novelty of the regulatory changes in the field of psilocybin therapy and the limited availability of large-scale clinical trials, most professional healthcare organizations and regulatory colleges have yet to establish such important standards. In their absence, physicians must gather their information from a variety of sometimes conflicting sources developed for different drugs or contexts that may not be completely objective (producers’ leaflet, advocacy notes, etc.). This is a far from ideal situation when considering the risks for patient’s safety and concomitant medical liability of the physician. It should also be noted that there is currently no official licencing or accreditation mechanism in Canada for those who wish to facilitate psilocybin therapy sessions.^57^ Much of the existing knowledge about psilocybin and other psychedelics comes from the context of medical research. Research is however intended to develop generalizable knowledge while ensuring a favorable benefit-risk ratio for participants. This raises the question of how guidelines developed for research should be adapted to the clinical care setting, where the priority is individual patient care and well-being. Perhaps existing studies on the relevant skills for therapists in psychedelic research could serve as a valuable starting point to assess the reasonableness of physicians’ conduct in the clinical setting.^58^ While not specific to psilocybin, within the field of psychedelic medicine, the College of Physicians and Surgeons of Alberta (CPSA) has issued a Statement on physician experiential learning for psychedelic medicines, taking the position that any physician participating in or supporting experiential learning in this context must accept “full professional responsibility and accountability for its use and any associated outcomes.”^59^ The CPSA additionally refers individuals to its standard for “Practising Outside of Established Conventional Medicine” which outlines key principles members should uphold, such as “always acting within the scope of their practice based on their qualifications, skill, knowledge and level of competence.”^60^ Importantly, according to the Standard, a conventional medical approach must be offered before suggesting any therapy outside of conventional medicine.^61^ Another potentially helpful standard bears on the use of ketamine for the treatment of depression. Although ketamine is not classified as a classical psychedelic, it is alike to psilocybin in that it possesses similar therapeutic applications and antidepressant effects as well as “dreamlike and hallucinogenic properties.”^62^ In 2021, the Canadian Network for Mood and Anxiety Treatments (CANMAT) issued guidelines on the use of racemic ketamine in adults with a major depressive disorder.^63^ While knowledge can be gained from other psychedelic treatments for developing standards specific to psilocybin, the differences between various modalities must be considered, such as their distinct effects and pharmacological sites of action.

Moving forward, clinical guidelines and specific standards regarding indications for psilocybin, dosage, administration, and follow-up should be developed to foster a more predictable practice surrounding psilocybin treatment.^64^ As standards are developed, steps should also be taken to promote specialized psilocybin therapy training for therapists, including graduate or certificate courses,^65^ such as that at the University of Ottawa,^66^ Vancouver Island University^67^ or the California Institute of Integral Studies.^68^ The development of standards can also assist in the process of credentialing and licensing to promote minimum standards of safety and accountability.^69^ Several non-governmental organizations (such as TheraPsil, the Michener Institute, and ATMA) have already begun offering training sessions to educate physicians about psilocybin treatment and to provide them with certificates.^70^ It would be highly beneficial for a wider range of organizations, bodies, and academics to engage in the process of developing standards of practice to better ensure the safety, efficacy, and fairness of psilocybin therapy. Tardy and deficient guidelines could be detrimental to the public perception of psychedelic medicine and potentially perilous to public health. In this early and critical stage in the development of psilocybin treatment, openly sharing information and practices from these training programs can accelerate the development of psilocybin treatment and promote the harmonization of practices locally, nationally, and internationally. A possible obstacle to harmony is a disagreement on what training for physicians should look like. One such point of divergence regards the necessity for physicians to have personal experience with psilocybin treatment, with some academics and commentators supporting mandatory experiential training^71^ and some training programs including such experiential components,^72^ while other authors suggest is it unethical to require firsthand experience.^73^ Another obstacle to the development of training is linked to the lack of clarity surrounding the appropriateness and framing of psychological supports for psilocybin treatment. Some promote a more basic psychological support while others encourage more intensive psychotherapy.^74^

Developing medical standards for psychedelics comes with open questions concerning the importance of relying on Indigenous communities’ existing frameworks for these practices. Indigenous communities’ use of psilocybin for centuries is a potential resource to be considered in the development of medical standards. Yet questions remain around the appropriateness of transplanting knowledge rooted in spiritual, ritualistic, and communal healing practices into the Western scientific model. Such an issue is particularly relevant in the current context of reconciliation and diversity existing in Canada.^75^ Several important steps can be taken to better promote an ethical practice of psilocybin therapy. There is a growing recognition among psychedelic therapy advocacy groups that practitioners and the public at large should be informed of Indigenous traditions and practices associated with psilocybin, and the associating worldview that motivates such practices, to develop a more holistic and effective practice. It is worth noting that some existing training, including those offered by TheraPsil,^76^ the Michener Institute,^77^ ATMA,^78^ as well as expert literature,^79^ include components on Indigenous teachings. Second, the process of developing guidelines on psilocybin research and practice should take note of the historically exploitative and unsustainable foraging for these psychedelic medicines. Third, any benefits of psilocybin research and treatment should be made equally available to Indigenous peoples. Finally, Indigenous scholars, knowledge holders, and practitioners should be actively included in the process of developing ethical and practical standards surrounding psilocybin therapy, in a way which promotes the self-determination of Indigenous Nations.^80^

## Medical Malpractice Liability

### Exploratory Vignettes: Situations Where Liability Issues Could Arise

In starting this discussion, we note that not all the situations described below are constitutive of medical malpractice liability. Some will instead constitute unethical medical practices from a therapist that could be addressed through professional organizations, health institutions of the provider, healthcare providers, etc. Finally in some cases, an adverse outcome may not be clearly explained by a legal or ethical fault from the therapist. These unfortunate situations would not be a source of ethical or legal liability. We believe well-developed standards of practice could help significantly reduce all these scenarios. Consider the actions, omissions, medical responsibility and potential fault of the practitioners in each of these vignettes. We suppose that each scenario takes place in Canada.

#### Situation 1: A doctor refuses to provide psilocybin to a patient that qualifies

*Georgina, recently diagnosed with a case of terminal metastatic breast cancer, has been suffering from treatment-resistant depression and end-of-life anxiety for the past few months with significant impacts on her quality of life. She has been reading about psilocybin treatment and would like to try it, as all other therapeutic options available or suitable to her have failed to appease her symptoms. At her regular appointment with her psychiatrist, Dr. Xavier, she asks him to help her apply to the Special Access Program. Dr. Xavier, unwilling to apply to the Special Access Program due to a lack of knowledge about psilocybin treatment and “not wanting to see his image tarnished by an association with psychedelics,” refuses. As she lives in a rural area, Georgina has no other psychiatrist to consult, and Health Canada has not replied to her application for a Section 56 exemption, leaving her with no other option to legally access psilocybin treatment. She is unable to afford to pay for it privately, with the cost of psilocybin treatment being prohibitively high. Georgina is left with inadequate treatment for her end-of-life anxiety.*

#### Situation 2: A patient claims he has been harmed following psilocybin assisted therapy

*A 40-year-old patient with late-stage cystic fibrosis named Adam has recently been prescribed and administered psilocybin by his physician, Dr. Newer, as part of psilocybin therapy to combat treatment-resistant depression as part of his palliative care. A request through the Special Access Program was made to access the psilocybin. After administering the psilocybin, Adam suffered increased anxiety and developed hallucinogen-persisting perception disorder (HPPD), which has been persisting for several months until the present. Upset with Dr. Newer, Adam claims that the prescription for psilocybin and the therapy session has caused him psychological damage. He decides to pursue a malpractice claim against Dr. Newer, alleging that her actions were inappropriate and that she was negligent in prescribing psilocybin and in carrying out the therapy session.*

#### Situation 3: A patient claims he has been harmed due to unethical psychotherapy practices during the psilocybin session

*A 25-year-old patient named Bachar recently underwent a psilocybin session as part of psilocybin therapy to help treat his anxiety disorder. As part of the psilocybin session, his prescribing psychiatrist, Dr. Smith, as well as a therapist, Dr. Johnson, working under Dr. Smith, helped to guide his session. Bachar was asked as part of the informed consent process whether he would like to consent to reassuring touch, if needed during the psilocybin session. Bachar consented to be touched. During the session, Bachar appeared to be in distress, and Dr. Johnson decided to offer reassuring touch to Bachar by gently touching his shoulder and holding his hand. There was no overt request for therapeutic touch from Bachar. After the session, Bachar claimed that that he was inappropriately touched by Dr. Johnson and that this led to an overall unpleasant and negative psilocybin experience. He is also worried about potential abuse(s) that may have happened during the session that he doesn’t remember now. He decides to pursue a malpractice claim against Dr. Smith, as Dr. Johnson was under his supervision. He also files an administrative complaint to the medical association of Dr. Smith.*

### Addressing medical liability issues

In negligence cases, courts assess whether the conduct of the alleged wrongdoer fell short of what a “reasonable person” would have done in the circumstances. In medical liability cases, a subset of negligence, courts assess the conduct of the medical expert against that of other experts possessing a reasonable level of knowledge or skill in that field.^81^ This standard is usually established, in a specific situation, through experts’ testimony and a review of existing standards of practice.^82^ Unless a professional standard is “fraught with obvious risk”^83^ or demonstrably unreasonable,^84^ adherence to it should obviate malpractice claims. Unclear, contradictory, or non-existent medical standards may prevent physicians from relying on courts’ usually deferential attitude to medical standards. Where the practitioner’s conduct fails to adhere to medical standards or where a practitioner adheres to demonstrably unreasonable guidelines, courts will conclude that the practitioner acted unreasonably and is exposed to liability. The law in the US in this regard is extremely similar to that in Canada.^85^ With standards for psilocybin treatment still in development, malpractice liability poses a real risk and can be a source of reluctance for those working in the field. One of the benefits of psilocybin — its long-lasting effects — can also exacerbate potential liability fears for physicians. With psilocybin therapy involving an integrative phase and follow-up care, physicians are potentially responsible for patients for an extended period of time, meaning that there may be a longer period where medical professionals need to follow up on their patient’s health and could incur malpractice suits for not doing so.

It is important to consider how the mechanism chosen to access psilocybin may impact the potential liability of the physician. The important distinction in this context is whether psilocybin is obtained for the purpose of a clinical trial (for research) or for clinical practice. When a practitioner obtains psilocybin for clinical practice, the choice of mechanism to access psilocybin will not alter their liability for malpractice. Thus, in Canada, whether the Special Access Program or a section 56 exemption is used to access psilocybin will not have an impact on the resulting liability of the practitioner. Nonetheless, it is important to identify the potential differences in how liability is assessed in the clinical trial setting as opposed to clinical practice. As academics note, the means of assessing the liability of a medical researcher is subject to some controversy.^86^ As with clinical practice, researchers must secure informed consent to carry out psilocybin treatment. Researchers must provide a full disclosure of what a reasonable participant would want to know in the circumstances; the duty of disclosure is “as great, if not greater than, the duty [of disclosure] owed by the ordinary physician.”^87^ The failure to properly obtain informed consent will lead to claims for battery and, in Canada, negligence.^88^ Nonetheless, obtaining consent does not obviate negligence. While there has been some American case law suggesting that the assessment of the conduct of a physician carrying out research is guided by what a reasonable *lay person* would do, it is generally accepted that physician researchers are assessed on the basis of a reasonable *physician.* ^89^ Therefore, where a physician carrying out a clinical trial fails to adhere to the standard of conduct that would be expected of a reasonable physician, they will be liable. As with the clinical practice setting, standards of practice and guidelines can help determine what a reasonable physician would have done.

As highlighted earlier, many people using psilocybin therapy are in positions of increased susceptibility to harm due to underlying conditions. The common law doctrine of the thin skill rule or “take your victim as you find them”, also imported into Quebec civil law, could make physicians liable for the full extent of patients’ injuries even where they are abnormally severe because of preexisting vulnerabilities.^90^ The thin skill rule prevents a practitioner from raising the defense that their victim was inordinately susceptible to suffering extensive injuries. Just because most people would have suffered no or minimal harm from a certain negligent act does not let the practitioner off the hook for extensive injuries.

In Quebec, recovering damages for negligently inflicted psychological injury has not been a significant problem,^91^ with courts consistently granting damages for such injuries.^92^ By contrast, the common law tradition has historically been reluctant in permitting recovery of damages for psychological injuries,^93^ but this is increasingly changing.^94^ Canadian common law provinces will now allow the recovery of a non-physical injury when it is serious and prolonged,^95^ but more than mere emotional upset.^96^ Common law provinces also no longer require plaintiffs to demonstrate a recognizable psychiatric illness.^97^ This brings greater harmony between Canadian common law’s position on recovery for psychiatric injury and that for physical injury.^98^ Thus, psychiatric injury is a recoverable injury across Canadian provinces which physicians may be forced to compensate.

Aside from negligence claims, physicians must also be aware of claims for battery. If touching or restraining becomes necessary during a psilocybin therapy session because of a patient’s agitation, physicians put themselves at risk of liability for the tort of battery and potential criminal liability.^99^ In the context of Quebec private law, similar risks could fall within the obligation to act reasonably under article 1457 of the Civil Code of Quebec. Many sources also raise awareness about the serious risk of sexual violence in context of psychedelic assisted therapies, and the potential resultant liability.^100^ The risk of sexual abuse is particularly prominent given the intense vulnerability and power imbalances inherent to psychedelic treatments. Instances of sexual abuse are also well documented in ayahuasca healing (an Amazonian spiritual healing practice using ayahuasca vine and chacruna) and have led to the development of guidelines targeted specifically at educating those in the psychedelic community about sexual abuse and prevention mechanisms.^101^ Guidelines targeting the prevention of sexual abuse would also be desirable for psilocybin treatment. Such guidelines should clearly prohibit any sexual contact or intimacy with the participant during or following the treatment. Guidelines ought to clearly delineate the proper boundaries of reassuring touch by prohibiting any contact which may be deemed sexual in nature and ensure this is sufficiently outlined during the informed consent process. Some also advocate for a male-female therapist pair to be always present during the psilocybin treatment sessions to reduce the likelihood of inappropriate behavior from a therapist with a patient.^102^

The legal doctrines of *respondeat superior*, vicarious liability, and their Quebec equivalent of “fault of another” are important to consider. Under these doctrines, employers and supervisors can be held liable for the acts of their employees or subordinates when in the scope of employment.^103^ Thus, supervising clinicians, who may not be present during long psilocybin treatment sessions, can be held liable for wrongs committed by the therapists or other medical professionals present. With psilocybin treatment lacking licensing, credentialing, and standardized training, supervisors are also at an increased risk of vicarious liability. This problem is particularly relevant in the US, considering that the first statewide psilocybin treatment service in Oregon does not require facilitators to have a college degree or advanced education.^104^ All of this may contribute to an unease in psychiatrists as they decide whether to facilitate or prescribe psilocybin treatment.

Returning to the three exploratory vignettes, it may be beneficial to outline how the presence of standards would protect the treating physician and benefit the patient. In Situation 1, the development of standards could destigmatize psilocybin treatment, leading to a greater willingness of Dr. Xavier to apply to the Special Access Program and the presence of more physicians carrying out psilocybin treatment in rural areas close to Georgina. This scenario illustrates that the public perception of psilocybin as well as the mere risk and uncertainty surrounding liability can impact health outcomes. In Situation 2, psilocybin treatment standards on inclusion/exclusion criteria may, though not necessarily given the uncertainty surrounding HPPD’s causes, aid in reducing its incidence. Adherence to those standards would allow Dr. Newer to avoid medical liability for prescribing and administering psilocybin to Adam. It would also be crucial in the informed consent process to outline the risk of HPPD and the uncertainty surrounding its causes, thereby reducing the likelihood of a battery claim. In Situation 3, standards on consent and reassuring touch would clarify the reasonableness of Dr. Johnson’s actions and allow patients like Bachar to better learn about psilocybin treatment prior to his session. The development, or adaptation, of existing sexual abuse guidelines could also allow Dr. Smith to implement effective anti-abuse training for therapists under his supervision to reduce the likelihood of vicarious liability.

## Conclusion

Given the current societal debate surrounding the increasing use of MAiD in Canada,^105^ it is becoming obvious that physicians should consider all potential alternative treatments to alleviate the physical and mental pain of end-of-life patients. One such treatment, using psilocybin, is getting increasingly positive support due to growing evidence of benefits in the research setting. Consequently, psilocybin treatment is now accessible through clinical trials, s. 56 CDSA exemptions, the Special Access Program, or through psychiatrists in Alberta. While access to psilocybin treatment remains limited, the trend towards loosening restrictions and the ongoing psychedelic renaissance warrants deeper investigation into the psilocybin’s potential.

With growing attention towards psilocybin treatment, the associated risk of medical liability for physicians involved in prescribing psilocybin or facilitating psilocybin treatment becomes increasingly important to study. This paper has highlighted the risks of liability that are present in the field. The lack of established medical standards, licensing, standardized training, and credentialing contributes to the heightened uncertainty and legal risks surrounding psilocybin treatment. This may contribute to fears among medical professionals concerning psilocybin treatment, which hinders access to this therapeutic modality. More clinical trials and meta-analyses of research will assist in the development of training and medical standards to help realize psilocybin’s potential. A focus on knowledge sharing throughout this developmental process will ensure harmony and efficacy in efforts to make psilocybin treatment a safer and more accessible practice.
